# Supplementary material for: Hydrocarbon Sorption in Flexible MOFs—Part III: Modulation of Gas Separation Mechanisms
Source: Nanomaterials (Basel). 2024 Jan 23;14(3):241. doi: 10.3390/nano14030241 (PMC10856790; doi:10.3390/nano14030241)
Supplement: Supplementary file 1 [file nanomaterials-14-00241-s001.zip › nanomaterials-2804327-supplementary.pdf]

Supplementary Materials

Hydrocarbon Sorption in Flexible MOFs—Part III: Modulation of Gas Separation Mechanisms

Hannes Preißler-Kurzhöfer [1,2,*], Marcus Lange [2], Jens Möllmer [2], Oliver Erhart [3], Merten Kobalz [3], Harald Krautscheid [3] and Roger Gläser [2,*]

[1] Institut für Technische Chemie, Fakultät für Chemie und Mineralogie, Universität Leipzig, Linnéstraße 3, D-04103 Leipzig, Germany
[2] Institut für Nichtklassische Chemie e.V., Universität Leipzig, Permoserstraße 15, D-04318 Leipzig, Germany; lange@inc.uni-leipzig.de (M.L.); moellmer@inc.uni-leipzig.de (J.M.)
[3] Institut für Anorganische Chemie, Fakultät für Chemie und Mineralogie, Universität Leipzig, Johannisallee 21, D-04103 Leipzig, Germany; oliver.erhart@uni-leipzig.de (O.E.); merten.kobalz@uni-leipzig.de (M.K.); krautscheid@rz.uni-leipzig.de (H.K.)
* Correspondence: che09bcz@studserv.uni-leipzig.de (H.P.-K.); roger.glaeser@uni-leipzig.de (R.G.)

S1. Isotherm fitting and gate-opening boundaries

Within this work, a dual-Dubinin-Asthakov equation (dual-DA) suitable for inhomogeneous adsorbents [1] with two adsorption spaces was applied to model all experimental isotherms, as was done in previous works [2,3].

$$W = W_{0,1}\, e^{-\left(\frac{A}{E_1}\right)^{m_1}} + W_{0,2}\, e^{-\left(\frac{A}{E_2}\right)^{m_2}} \tag{1}$$

Herein, W_0 is the saturation loading, while E and m mark the specific adsorption energy and the inhomogeneity parameter respectively. Since it is a dual-site model, all three parameters are denoted with either 1 or 2.

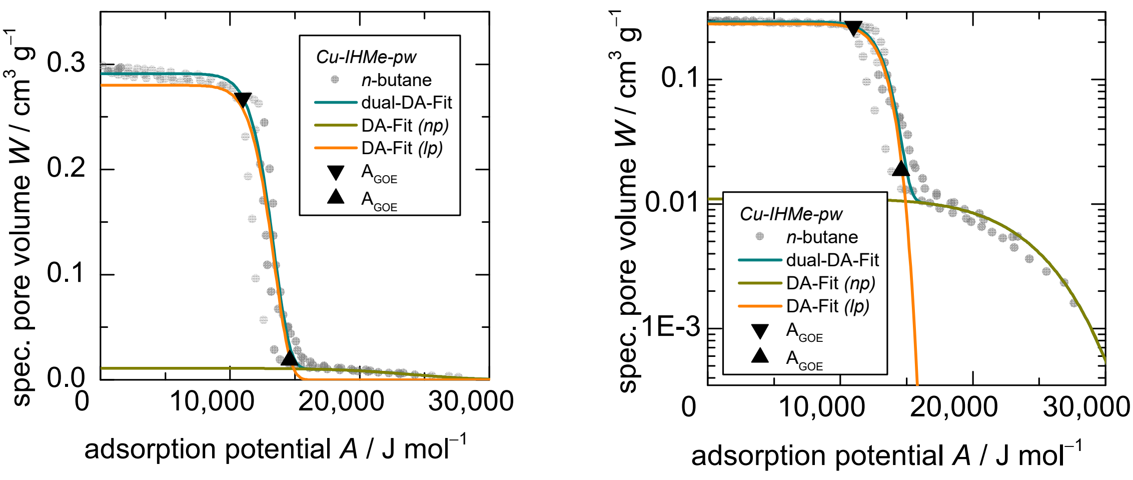

Figure S1: Fitting approach using the dual-DA equation for *n*-butane on *Cu-IHMe-pw* and the resulting gate-opening boundaries.

After fitting, essential points within the isotherms can be specified. For flexible MOFs, it is common that one gate-opening pressure is defined. However, due to particle size distributions of the adsorbents, different energy barriers for the structural transition can occur and therefore, pressure ranges rather than isobaric phase changes are observed. Thus, in this work, two pressure points are defined to describe the entire gate-opening. For flexible MOFs, the first term in the dual-DA equation (1) describes the sorption in the narrow pore phase (*np*-phase), while the second term describes the structural transition and medium pore phase (*mp*-phase) sorption. The gate-opening starts when the latter term starts adding significant pore volume. Therefore, to find a gate-opening start (GOS) point in the isotherm, a boundary value of 0.01 $cm^3\ g^{-1}$ for the *mp*-phase term was set.

Secondly, a gate-opening end (GOE) is determined, herein defined using the excess surface work (ESW). The ESW, usually computed as the product of the adsorbed amount and the change in chemical potential, gives rise to the strength of interaction between the sorbent and the sorptive according to Adolphs [4]. It is mathematically identical to the first derivative of the surface potential as defined by Myers [5]. It describes the counter acting processes of decreasing surface free energy and increasing isothermal and isobaric work of sorption. The occurring minimum in the plots can then be identified as the completion of a monolayer. In this work, the product of the adsorption potential and the volumetric loading was used to identify the position of a quasi-monolayer at an ESW-maximum (see Fig. S2). It has to be stated that the resulting ESW-values are not of relevance in this case but just the

position of the extrema. Analogous to the adsorption, the desorption patterns can be fitted as well and subsequently the gate-closing start (GCS) and gate-closing end (GCE) points derived.

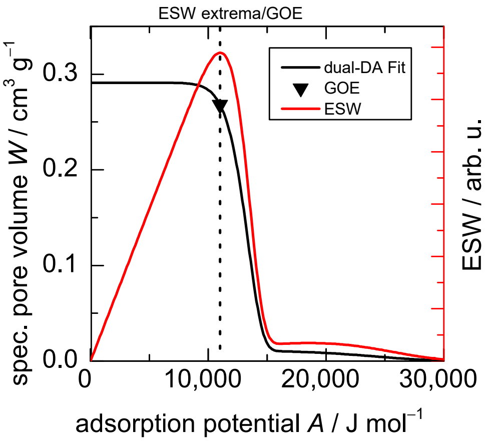

Figure S2: Determination of gate-opening end (GOE) using the excess surface work (ESW) theory.

Furthermore, a new isotherm analysis methodology, the Dubinin-based universal adsorption theory or *D-UAT* were recently published [2]. It utilizes the corresponding states theory by diving the adsorption potential by the critical temperatures of the gases. This leads to a normalisation of the gas state and allows just comparisons of the reduced interaction potentials which are independent of the gas properties. The fits for the ad- and desorption of all system investigated in this work are presented in Figure S3, the reduced interaction potentials in dependence of sorptive loading are shown in Figure S4, the fitting parameters are presented in Table S1.

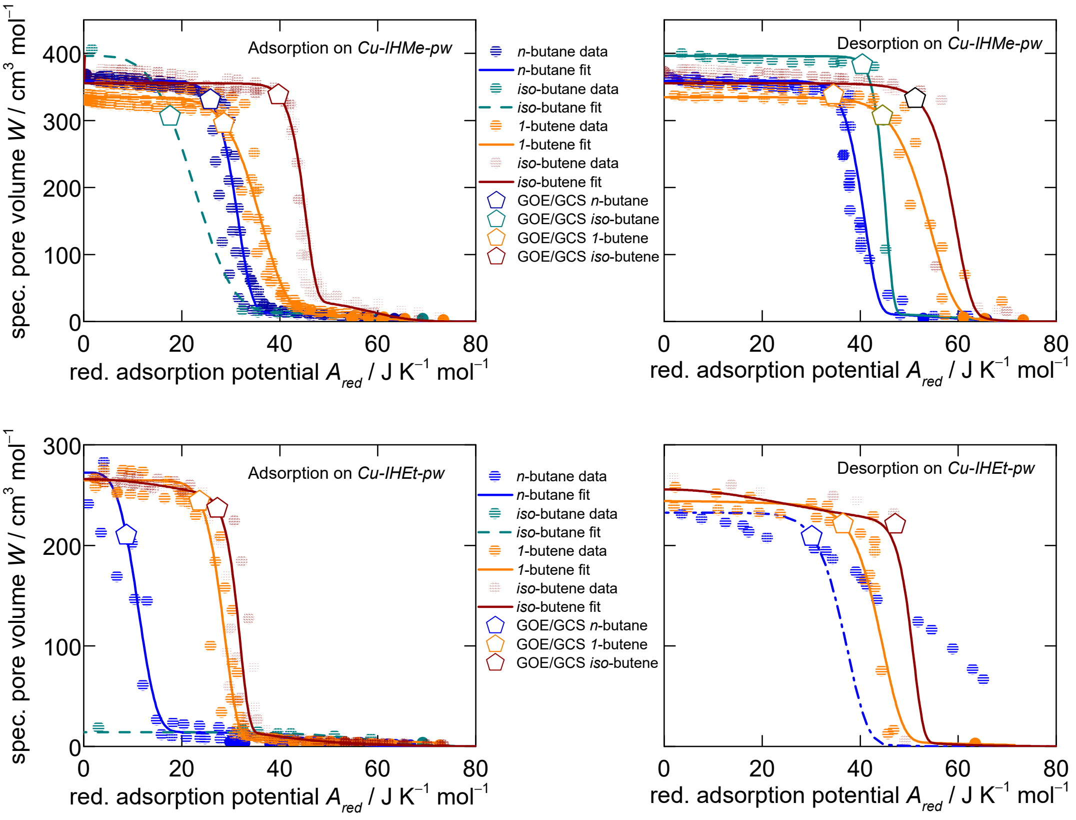

Figure S3: Characteristic reduced patterns for adsorption (left) and desorption (right) after the application of the D-UAT of the C4-hydrocarbons *n*-butane, *iso*-butane, *1*-butene and *iso*-butene on *Cu-IHMe-pw* (top) and *Cu-IHEt-pw* (bottom) measured at temperatures of 283, 298 and 313 K. Please note that iso-butane was not able to open the framework within *Cu-IHEt-pw* and thus is omitted in the lower right-hand graph.

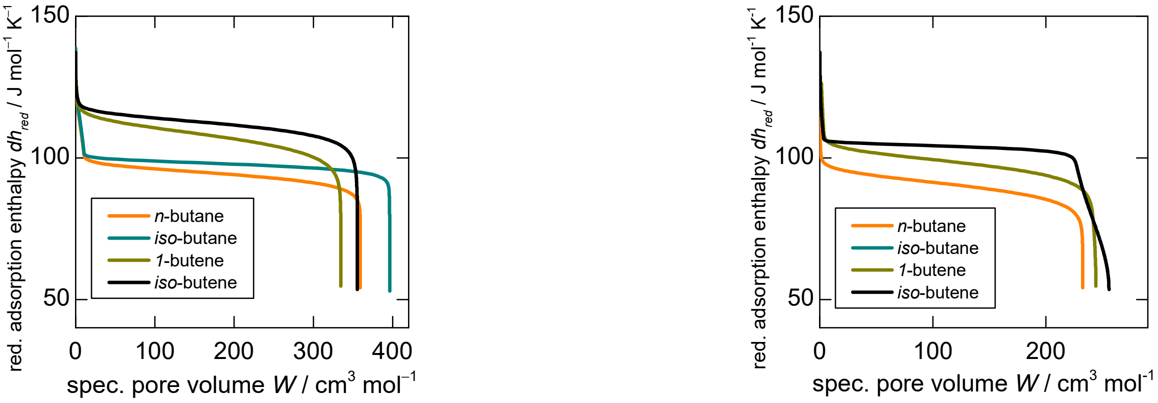

Figure S4: Reduced enthalpy of adsorption derived from the dual-Dubinin-Asthakov Fits of the desorption patterns and the D-UAT for the C4-hydrocarbons on *Cu-IHMe-pw* and *Cu-IHEt-pw*. Please note that iso-butane was not able to open the framework within *Cu-IHEt-pw* and thus is omitted in the right-hand graph.

Table S1: Reduced fitting parameters for the dual-Dubinin-Asthakov model (dual-DA-Fit) ad- and desorption for all C4 adsorptives in both flexible MOFs.

MOF	Process	Adsorptive	Pore phase *i*	$W_{0,i}$ / $cm^3 g^{-1}$	E_i / J $mol^{-1} K^{-1}$	m_i
Cu-IHMe-pw	ADS	*n*-butane	*np*-phase	0.011	-59	6
			mp-phase	0.28	-32	12
		iso-butane	*np*-phase	0.011	-61	6
			mp-phase	0.32	-25	4
		1-butene	*np*-phase	0.011	-60	6
			mp-phase	0.26	-37	8
		iso-butene	*np*-phase	0.028	-60	8
			mp-phase	0.26	-45	22
	DES	*n*-butane	*np*-phase	0.011	-59	6
			mp-phase	0.28	-41	16
		iso-butane	*np*-phase	0.011	-61	6
			mp-phase	0.31	-45	30
		1-butene	*np*-phase	0.011	-60	6
			mp-phase	0.26	-55	12
		iso-butene	*np*-phase	0.028	-60	8
			mp-phase	0.26	-60	4
Cu-IHEt-pw	ADS	*n*-butane	*np*-phase	0.011	-50	6
			mp-phase	0.2	-12	4
		iso-butane	*np*-phase	0.011	-61	6
			mp-phase	-	-	-
		1-butene	*np*-phase	0.005	-80	6
			mp-phase	0.2	-29	12
		iso-butene	*np*-phase	0.028	-35	2
			mp-phase	0.178	-32	18
	DES	*n*-butane	*np*-phase	0.011	-50	6
			mp-phase	0.2	-60	2
		iso-butane	*np*-phase	-	-	-
			mp-phase	-	-	-
		1-butene	*np*-phase	0.009	-50	2
			mp-phase	0.18	-45	12
		iso-butene	*np*-phase	0.028	-35	2
			mp-phase	0.17	-51	26

S2. Kinetic analysis

The kinetics of an adsorption process are pre-dominantly analyzed via two approaches. The analysis of large pressure steps and the subsequent fitting of the uptake curves give a general overview over the governing features during this process, e.g pore width limitations, surface-barriers or no*n*-isothermal behavior. The relevant fitting approaches are explained within the next section. Secondly, there is the opportunity to measure gas uptake for small pressure steps in order to calculate the resulting transport diffusivities in dependence of adsorptive loading. For such measurements, the IR-microscopy method proposed by Chmelik [6] has been established in recent years for delivering reliable information due to its fast data acquisition and independence of scale movements. However, due to the very prolonged timeframes of adsorption during the gate-opening, the utilization of this method was not feasible. Thus, the gravimetric uptakes during the measurements of the adsorption isotherms were analyzed in the same way, which showed similar patterns for the resulting transport diffusivities (see Figure S5). The data points themselves deviate by a factor between 2 and 3, which was deemed acceptable for the purpose of estimating the overall mechanism of the structural transition.

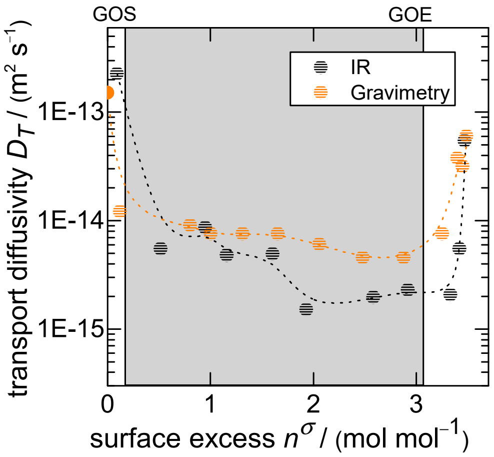

Figure S5: Transport diffusivity measurements via IR-microscopy [2,6] and gravimetry for the adsorption of *n*-butane on *Cu-IHMe-pw* at 298 K.

S3. Static Gas-Mixture Measurements and Model Development

S3-1 Detailed description of measurement set-up and approach

As outlined in the main manuscript, a self-built hybrid gravimetric-manometric apparatus was used based on the previous works of Lange [7], with Fig. S6 depicting the overall set-up. Herein, the manometric part consists of several gas vessels (Vorlage 1, 2 and 3) with valves V1 to V11 able to separate different sections within the apparatus. Furthermore, two gas-inlets and the connection to a vacuo-pump (TMP) is part of the setting which is directly connected to the suspension balance (MSW).

Prior to each measurement, the exact amounts required for the experiment were calculated via a mass balance, incorporating the overall available volume within the apparatus, the assumed adsorbed amounts per component as well as temperature. The adsorbent itself was evacuated within the suspension balance with the opening of valve V8. The individual gas-components were being placed within the gas vessels Vorlage 1, 2 or 3 with pressure control. The individual chambers were then connected via the opening of the valves V6, V10 and V9 and the subsequent gas mixture homogenized using a circulating pump. After complete homogenization of the mixture monitored via GC-FID measurements, valve V8 was closed and V11 opened, allowing the mixture to enter the suspension balance and the evacuated adsorbent. The development of the equilibrium was monitored by the weight change of the magnetic suspension balance as well as by the constancy of the gas phase composition determined by GC-FID analyses.

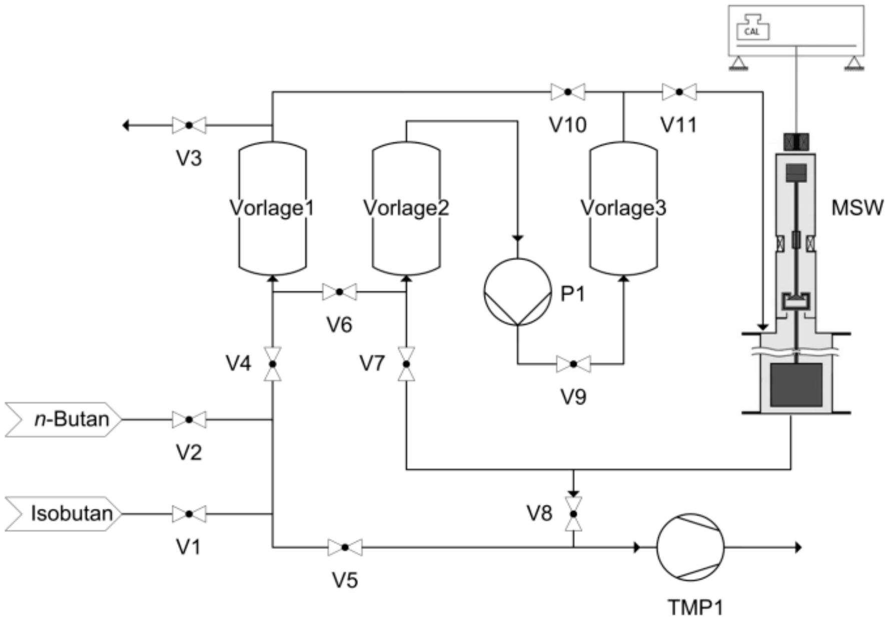

Figure S6: Illustration for the hybrid gravimetric-manometric measurement set-up for binary gas mixture, based on [7]. Herein, V1 to V11 are valves, Vorlage 1-3 are gas vessels used to prepare the gas mixtures.

S3-2 Gas-uptake model and mass balance

As outlined in the previous section, the results of the gas mixture experiments are a gravimetrically measured overall gas-uptake on the adsorbent as well as the composition of the gas-phase measured via GC-FID. Within a second step, these results were modelled within an overall mass balance in order to calculate the composition of the adsorbed phase and the overall separation selectivity.
For the fitting of the overall gas-uptakes of the gas-mixtures, the "GO"-model developed by Tanaka et al. was used [8]. Although it was recently shown [3] to not be able to mimic all the idiosyncrasies present in the dynamic uptake of flexible MOFs, the model shows generally good agreements with experimental data. In its core, the model deploys two different rate-constants, one for the description of the diffusion processes k_D while the second is concerned with the rate of structural transition k_{GO}. In our recent study specifically concerned with the overall features governing the kinetics of gas uptakes in flexible MOFs, an additional diffusion rate was implemented in order to account for outer-surface adsorption. This becomes necessary especially for uptakes very close to the overall equilibrium of the structural transition. Since such uptakes are not the focus of this work, this step was omitted herein and the same mathematical formulation used as originally outlined by Tanaka for the "GO"-model:

$$F(t) = \left\{1 - \frac{k_{GO}\exp(-k_D t) - k_D \exp(-k_{GO} t)}{k_{GO} - k_D}\right\}, \tag{2}$$

For the analysis of the gas mixture uptakes, the rate constant for diffusion k_D refers to the diffusion of the entire gas mix, regardless of the species. To model the evolution of gas-uptake for both species individually, a second formulation for the gas re-exchange of the slower species was assumed with a simple LDF-approach. Herein, it is assumed that the slower species (described by F_2) replaces the faster species (described by F_1) within the framework and thus allows the formulation:

$$F_2(t) = \Psi_2\,(1 - \exp(-k_{D-ex} t)), \tag{3}$$

$$F_1(t) = \left\{1 - \frac{k_{GO}\exp(-k_D t) - k_D \exp(-k_{GO} t)}{k_{GO} - k_D}\right\} - F_2(t), \tag{4}$$

Herein, Ψ_2 represents the fraction of uptake the second species has in the equilibrium or quasi-equilibrium. The parameter k_{D-ex} is much slower than k_D given the low rate of gas-exchange. While both k_{GO} and k_D can be fit from the uptake-data, the parameter k_{D-ex} is fit so the model represents the overall gas compositions in dependence of time measured via GC. The relevant fitting parameters are shown in Table S2.

Table S2: Fitting parameters for the gas mixture model for the three gas separations conducted in the main manuscript.

all in s^{-1}	*Cu-IHMe-pw*<br>*n*-butane / *iso*-butane<br>0 – 40 kPa	*Cu-IHEt-pw*<br>*n*-butane / *iso*-butane<br>0 – 200 kPa	*Cu-IHEt-pw*<br>*iso*-butene / *iso*-butane<br>0 – 20 kPa
k_{GO}	$2.5\cdot10^{-3}$	$1.0\cdot10^{-2}$	$1.0\cdot10^{-3}$
k_D	$1.5\cdot10^{-1}$	$4.0\cdot10^{-2}$	$4.0\cdot10^{-2}$
k_{D-ex}	$2.0\cdot10^{-4}$	$<2\cdot10^{-9}$	$<2\cdot10^{-9}$

The values are hard to compare given the different boundary conditions of the measurements. Although it was established within the main manuscript that the ability of gases to diffuse through the opened pore framework of *Cu-IHEt-pw* is much slower compared to *Cu-IHMe-pw* due to the differences in pore widths, the k_D parameter increases from measurement 1 to measurement 2. The same is observed for the parameter k_{GO}. This is caused by the much larger pressure step of the measurement 2 which overshoots the boundary potential of the structural transition much more than measurement 1. The largest difference between measurements on *Cu-IHMe-pw* and *Cu-IHEt-pw* can be seen in the gas exchange diffusion constant k_{D-ex}. Due to the narrow pore geometries, a gas exchange is not observable even after ~10,000 minutes (1 week), leading to the assumption that the exchange coefficient is at least lower than $2 \cdot 10^{-4}$ s^{-1}.
Generally, the model can be used as a basis for the analysis of static binary gas measurements in the future, helping to understand the governing features regarding the rate of structural transition, diffusion and gas-re-exchange.

S3-3 Detailed description of the overshoot potential

As outlined in the main manuscript, the overall driving force of the structural transition can be defined with some simplified assumptions as the potential difference between the set pressure point and the pressure point at the gate-closing start as given by the desorption pattern [3]. Generally, it would be assumed that the higher the driving force, the faster the gate-opening and thus overall gas-uptakes. This is mathematically described in formulation 5.

$$k_{GO} \sim dA_0 = A_{app} - A_{GCS} = RT \ln \frac{p_{app}}{p_{GCS}} \tag{5}$$

The formulation would only hold true if the respective loadings at the GCS and the applied pressure point are very similar. Within this section, this driving force and the further effect of the activation energy E_A as inhibitor will be accounted for in more detail based on energy profiles in order to generate a more detailed picture. Incorporating the molar loadings at these specific points, the overall absolute energy difference can be derived as E_O based on the integration of the respective sorption potential ranges to derive equation 6.

$$E_0 = E_{app} - E_{GCS} = \int_{\infty}^{A_{app}} N_{app} \cdot A_{app} - \int_{\infty}^{A_{GCS}} N_{GCS} \cdot A_{GCS} \tag{6}$$

Based on the formulations of Coudert and the grand canonical potential profile of a flexible MOF [9], at the gate-opening point both the narrow-pore and medium-pore phase have the same energy. Within the latter one, this is assumed to be the gate-closing start point. Therefore, E_0 represents the overshot potential that drives the phase transition towards the medium-pore phase. This is further illustrated within Fig. S 7.

However, as outlined in the main manuscript as well, the energy overshot for *iso*-butane is larger compared to *n*-butane based on the sorption isotherm patterns while the overall kinetics are much slower. The effect can potentially be ascribed to the necessary activation energy E_A of the gate-opening itself. This is the energy necessary for the molecular re-orientation of the frameworks intermediate state to occur and is sketched in Fig. S7 for both *n*-butane and *iso*-butane as it is not possible to derive this parameter from isotherms alone. Taking the analogy of the transition-state theory (TST) [10], the larger the activation energy, the unlikelier the structural transition becomes. This

leads to the conclusion that *iso*-butane very likely has a much larger activation than *n*-butane although is thermodynamically preferred under equilibrium conditions. This is probably caused by the bulkier nature of the sorptive and thus the greater spatial demand of it during the re-orientation of the framework itself. This however cannot be quantified from experimental data as such insights can only be gained by computational approaches. Therefore, the activation energies within the intermediate between the *np*- and *mp*-phase were only qualitatively ranked in order to illustrate the governing mechanism in Fig. S 7.

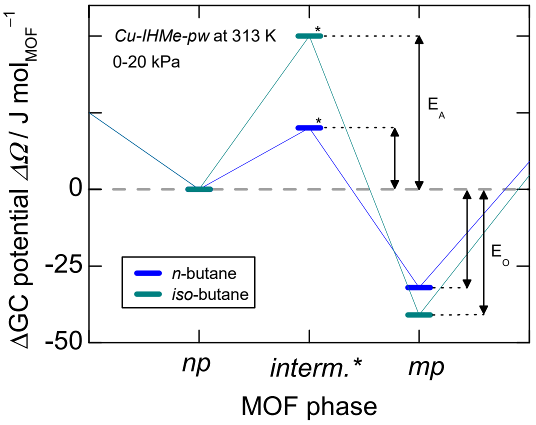

Figure S7: Illustration of the energy-profile at 313 K and 20 kPa for both *n*-butane and *iso*-butane on *Cu-IHMe-pw*. Herein, it is assumed that the energy of host and gas remains constant for the *np*-phase since no adsorption takes place. The change of energy for the *mp*-phase ($\boldsymbol{E_O}$) was calculated via the Dubinin-Asthakov-fits of the desorption isotherms. *The energy of the intermediate phases and the subsequent activation energies ($\boldsymbol{E_A}$) however are only qualitatively ranked based on the kinetic data.

Further Picture:

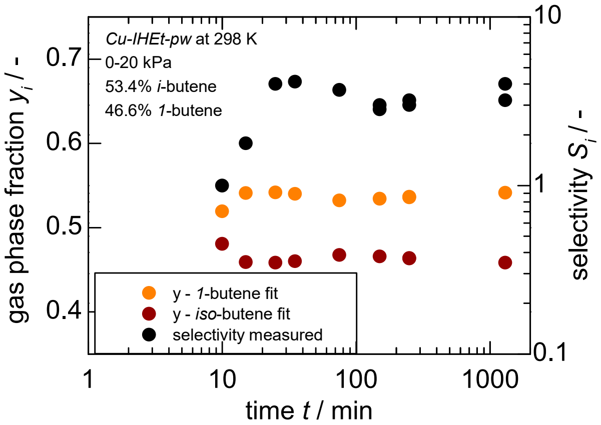

Figure S8: Gas-phase composition and separation factor in dependence of time for a “50:50”-mixture of *iso*-butene and *1*-butene on *Cu-IHEt-pw* for a pressure jump of 0-20 kPa.

S4 Additional Structural Data

Table S3 gives an overview over the relevant crystallographic data for the herein used MOFs and their individual phases. A description of the structure solution of the *mp*-phase of *Cu-IHMe-pw* can be viewed in [2].

Table S3: Crystallographic data of *[Cu_2(H-Me-trz-Ia)$_2$]* (both medium-pore and narrow-pore form) and of *[Cu_2(H-Et-trz-Ia)$_2$]*

Compound<br>Form	[Cu_2(H-Me-trz-Ia)$_2$]<br>Open *lp*-form	[Cu_2(H-Me-trz-Ia)$_2$]<br>*mp*-form	[Cu_2(H-Me-trz-Ia)$_2$]<br>Closed *np*-form	[Cu_2(H-Et-trz-Ia)$_2$]<br>Open *lp*-form*
Analysis method	single crystal data	crystal powder data	crystal powder data	crystal powder data
Molar Mass per UC / g mol^{-1}	1234.94	1234.94	1234.94	1291.04
Crystal system	monoclinic	monoclinic	monoclinic	monoclinic
Space group	$P\,2_1/c$ (no. 14)	$P\,2_1/c$ (no. 14)	$P\,2_1/c$ (no. 14)	$P\,2_1/c$ (no. 14)
Unit cell parameters / pm, °	a = 1085.9(1)<br>b = 1278.6(2)<br>c = 1402.6(2)<br>β = 110.508(8)	a = 1098.10(5)<br>b = 1498.83(5)<br>c = 1174.66(5)<br>β = 120.138(3)	a = 1077.90(5)<br>b = 1522.16(6)<br>c = 775.14(4)<br>β = 115.420(5)	a = 1089(1)<br>b = 1199(1)<br>c = 1459(1)<br>β = 109.9(1)
Volume / 10^6 pm^3	1823.9(4)	1672.0(1)	1148.7(1)	1793(3)
Z	2	2	2	2
Density / g cm^3	1.124	1.227	1.785	1.196
Porosity / %	50	43.3	4	39.9
Pore Volume / cm^3 g^{-1}	0.45	0.294	0.023	0.330
R_{int}	0.0585			
R_1	0.0587**			
wR_2	0.1597**			
R_p		0.0694	0.0607	
R_{wp}		0.0896	0.0750	

* The structure assignment of *Cu-IHEt-pw* is based on the unit cell parameters determined from X-ray powder diffraction data which are similar to those of the large pore form of *Cu-IHMe-pw*. [M. Kobalz, J. Lincke, K. Kobalz, O. Erhart, J. Bergmann, D. Lässig, M. Lange, J. Möllmer, R. Gläser, R. Staudt, H. Krautscheid, Inorg. Chem. 55 (2016) 3030–3039]

** The PLATON/SQUEEZE routine was used to remove the diffuse residual electron density as no solvent molecules could be localized during single-crystal structure refinement.

S5 References ESI

[1] Burevski, D. The application of the Dubinin-Astakhov equation to the characterization of microporous carbons. *Colloid & Polymer Sci* **1982**, *260*, 623–627, doi:10.1007/BF01422595.

[2] Preißler-Kurzhöfer, H.; Lange, M.; Kolesnikov, A.; Möllmer, J.; Erhart, O.; Kobalz, M.; Krautscheid, H.; Gläser, R. Hydrocarbon Sorption in Flexible MOFs-Part I: Thermodynamic Analysis with the Dubinin-Based Universal Adsorption Theory (D-UAT). *Nanomaterials* **2022**, *12*, doi:10.3390/nano12142415.

[3] Preißler-Kurzhöfer, H.; Kolesnikov, A.; Lange, M.; Möllmer, J.; Erhart, O.; Kobalz, M.; Hwang, S.; Chmelik, C.; Krautscheid, H.; Gläser, R. Hydrocarbon Sorption in Flexible MOFs-Part II: Understanding Adsorption Kinetics. *Nanomaterials (Basel)* **2023**, *13*, doi:10.3390/nano13030601.

[4] Adolphs, J. Excess surface work?: A modelless way of getting surface energies and specific surface areas directly from sorption isotherms. *App. Surf. Sci.* **2007**, *253*, 5645–5649, doi:10.1016/j.apsusc.2006.12.089.

[5] Myers, A.L. Thermodynamics of adsorption in porous materials. *AIChE J.* **2002**, *48*, 145–160, doi:10.1002/aic.690480115.

[6] Chmelik, C.; Kärger, J. The predictive power of classical transition state theory revealed in diffusion studies with MOF ZIF-8. *Micropor. Mesopor. Mat.* **2016**, *225*, 128–132, doi:10.1016/j.micromeso.2015.11.051.

[7] Tanaka, D.; Nakagawa, K.; Higuchi, M.; Horike, S.; Kubota, Y.; Kobayashi, T.C.; Takata, M.; Kitagawa, S. Kinetic gate-opening process in a flexible porous coordination polymer. *Angew. Chem. Int. Ed.* **2008**, *47*, 3914–3918, doi:10.1002/anie.200705822.

[8] Lange, M. Sorption von C4-Kohlenwasserstoffen and strukturell flexiblen, porösen Koordinationspolymeren. PhD-Thesis; Universität Leipzig, Leipzig, 2015.

[9] Coudert, F.-X.; Jeffroy, M.; Fuchs, A.H.; Boutin, A.; Mellot-Draznieks, C. Thermodynamics of guest-induced structural transitions in hybrid organic-inorganic frameworks. *J. Am. Chem. Soc.* **2008**, *130*, 14294–14302, doi:10.1021/ja805129c.

[10] Camp, J.S.; Sholl, D.S. Transition State Theory Methods To Measure Diffusion in Flexible Nanoporous Materials: Application to a Porous Organic Cage Crystal. *J. Phys. Chem. C* **2016**, *120*, 1110–1120, doi:10.1021/acs.jpcc.5b11111.

[11] Vanduyfhuys, L.; Rogge, S.M.J.; Wieme, J.; Vandenbrande, S.; Maurin, G.; Waroquier, M.; van Speybroeck, V. Thermodynamic insight into stimuli-responsive behaviour of soft porous crystals. *Nat. Commun.* **2018**, *9*, 204, doi:10.1038/s41467-017-02666-y.

[12] Tanaka, H.; Miyahara, M.T. Free energy calculations for adsorption-induced deformation of flexible metal–organic frameworks. *Curr. Opin. Chem. Eng.* **2019**, *24*, 19–25, doi:10.1016/j.coche.2019.01.001.

[13] Ghysels, A.; Vanduyfhuys, L.; Vandichel, M.; Waroquier, M.; van Speybroeck, V.; Smit, B. On the thermodynamics of framework breathing: A Free Energy Model for Gas Adsorption in MIL-53. *J. Phys. Chem. C* **2013**, *117*, 11540–11554, doi:10.1021/jp311601q.

[14] Kobalz, M.; Lincke, J.; Kobalz, K.; Erhart, O.; Bergmann, J.; Lässig, D.; Lange, M.; Möllmer, J.; Gläser, R.; Staudt, R.; et al. Paddle wheel based triazolyl isophthalate MOFs: Impact of linker modification on crystal structure and gas sorption properties. Inorg. Chem. 2016, 55, 3030–3039, doi:10.1021/acs.inorgchem.5b02921.
